# Phthalates and Mixed Alkyl Phthalates Analysis in Plastics Using GC‐MS With Cold EI

**DOI:** 10.1002/jms.70062

**Published:** 2026-04-24

**Authors:** Oneg Elkabets, Benny Neumark, Aviv Amirav

**Affiliations:** ^1^ Tel Aviv University Tel Aviv Israel; ^2^ Aviv Analytical Ltd Hod Hasharon Israel

## Abstract

Phthalates are esters of phthalic acid. They are widely used as plasticizers to improve the flexibility, durability, and performance of plastics in a variety of products. However, certain phthalates are associated with significant health risks, including endocrine disruption and reproductive toxicity. Consequently, regulatory agencies worldwide implemented restrictions on the use of phthalates, particularly in children's products. Gas chromatography–mass spectrometry (GC‐MS) is useful for phthalates analysis; however, heavy phthalates, such as diisononyl phthalate (DINP), diisodecyl phthalate (DIDP), and diundecyl phthalate (DIUP), present significant challenges due to their multiple isomers and absence of molecular ions using standard electron ionization (EI). These challenges are further compounded in cases of mixed alkyl phthalates, where existing methods fail to provide the essential molecular ions, hence cannot serve for their identification and quantification. Phthalate analysis is described with GC‐MS with Cold EI, which is based on the coupling of the GC and MS with a supersonic molecular beam and on electron ionization of vibrationally cold sample molecules (thereby named Cold EI) during their flight‐through a contact‐free ion source. This method offers a transformative solution for heavy and mixed alkyl phthalates analysis as Cold EI provides a clear molecular ion for unambiguous identification. Our phthalate analysis using GC‐MS with Cold EI is characterized by the following: (a) Plastic samples can be analyzed “as is” for their contents using ChromatoProbe for both sample introduction and thermal desorption; (b) identification and quantification of heavy and mixed alkyl phthalates are enabled via the provision of molecular ions and (c) much faster analysis.

## 1 Introduction

Phthalates are typically colorless, oily liquids semivolatile compounds [1]. Some phthalates such as di(2‐ethylhexyl) phthalate (DEHP) have a mild odor, whereas other species like dibutyl phthalate (DBP) or DINP may exhibit a faint aromatic or ester‐like scent [2]. They are produced by the esterification between an excess of alcohol (linear or branched) and phthalic anhydride in the presence of a catalyst [3, 4]. Phthalates are the most widely produced plasticizers, accounting for roughly 60% of the European plasticizer market and one third of global plastic additives [5] and are used extensively in the manufacture of plastic materials, mainly as PVC (polyvinyl chloride) [6, 7]. Their widespread use stems from a combination of desirable properties: They effectively reduce the rigidity of hard plastics — making them softer, more flexible and elastic. They also improve durability, transparency, and longevity, while enhancing processing characteristics such as flow, molding, and thermal stability [6, 7, 8]. These features make them highly valuable in a wide range of applications, including electric wires and cables, flooring, packaging materials, cosmetics, automotive parts, toys, and medical devices [9]. Phthalates are not chemically (covalently) bonded to the polymer matrix but are instead physically blended into it, which allows them to migrate out of materials and be released into the environment [9, 10, 11] or thermally desorb for their analysis. As a result, human exposure may occur through inhalation, ingestion, and dermal absorption [4, 10]. This raises concerns about potential health risks associated with phthalates. The main health risk is endocrine disruption. Phthalates can interfere with hormone signaling and have been associated with reproductive toxicity, including reduced testosterone levels, impaired sperm quality, altered development of the male reproductive tract, and disruptions in ovarian function and fertility [5, 9, 10, 12]. Such effects can lead to a variety of endocrine‐related health disorders, particularly when exposure occurs during critical developmental windows such as fetal development, infancy, and puberty [9, 10]. As a result, numerous countries and international bodies have implemented restrictions or bans on specific phthalates, particularly in products intended for children, but also in medical devices, food packaging, and cosmetics [8, 9, 10]. For example, the European Commission has classified several phthalates as substances toxic for reproduction under REACH (Regulation on the Registration, Evaluation, Authorization and Restriction of Chemicals) and has restricted their use in consumer products to a concentration below 0.1% by weight [13]. Similarly, the US Consumer Product Safety Commission (CPSC) has imposed the same limit (0.1%) on the use of certain phthalates on children's toys through the Consumer Product Safety Improvement Act (CPSIA) [14]. These regulations aim to reduce human exposure and reduce the potential health impacts associated with phthalate migration from plastic materials into the environment and to the human body.

Currently, phthalates are analyzed using various analytical techniques, including gas chromatography coupled with flame ionization detection (GC‐FID), electron capture detection (GC‐ECD), high‐performance liquid chromatography with tandem mass spectrometry (HPLC‐MS/MS), and gas chromatography‐Fourier‐transform infrared spectroscopy (GC‐FTIR) [15]. Among these, gas chromatography–mass spectrometry (GC‐MS) is considered the gold standard for phthalates detection due to its high sensitivity and specificity [11]. However, standard GC‐MS often fails to accurately identify complex mixtures of mixed alkyl phthalates, such as octyl‐nonyl or nonyl‐decyl isomers, due to the absence or low abundance of molecular ions in their electron ionization (EI) mass spectra. George and Prest [16] were the first to indicate this challenge and have clearly identified the presence of mixed alkyl phthalates using ammonia‐based positive chemical ionization on a technical DINP. Because molecular ions are essential for confirming molecular weight and distinguishing between structurally similar isomers, their absence significantly limits the ability of standard GC‐MS to accurately characterize and detect such compounds because their fragment ions do not relate to their mixed alkyl phthalate origin. In contrast, GC‐MS with Cold EI significantly enhances the molecular ions while retaining informative fragment ions and EI mass spectral library search compatibility such as NIST EI mass spectral library [17]. This is achieved through the ionization of vibrationally cold molecules and compatibility with high column flow rates (up to 32 mL/min), enabling confident monitoring, identification, and quantification of mixed alkyl phthalates that are otherwise impossible to resolve [17, 18]. Additionally, Cold EI improves all the major aspects of GC‐MS performance, including faster analysis, an extended range of analyzable compounds, uniform analyte response, and enhanced sensitivity and selectivity [17, 18, 19]. Moreover, the analysis of plastics for the detection of phthalates requires an extended process of sample preparation, which includes extraction using solvents, clean up using sorbents to remove interferences, concentration, and filtration [15]. In this study, we demonstrate the analysis of plastics using a ChromatoProbe (also known as the thermal separation probe from Agilent) for sample introduction and thermal desorption [20, 21]. This allows minimal and simple sample preparation, which merely requires cutting a small piece (below 1 mm^3^) of the plastic and placing it inside the ChromatoProbe vial for its thermal extraction, thereby eliminating the need for lengthy pretreatment steps.

This paper presents an efficient streamlined method for analyzing phthalates and mixed alkyl phthalates in plastic materials using ChromatoProbe sampling and GC‐MS with Cold EI.

## 2 Experimental

**FIGURE 1:**
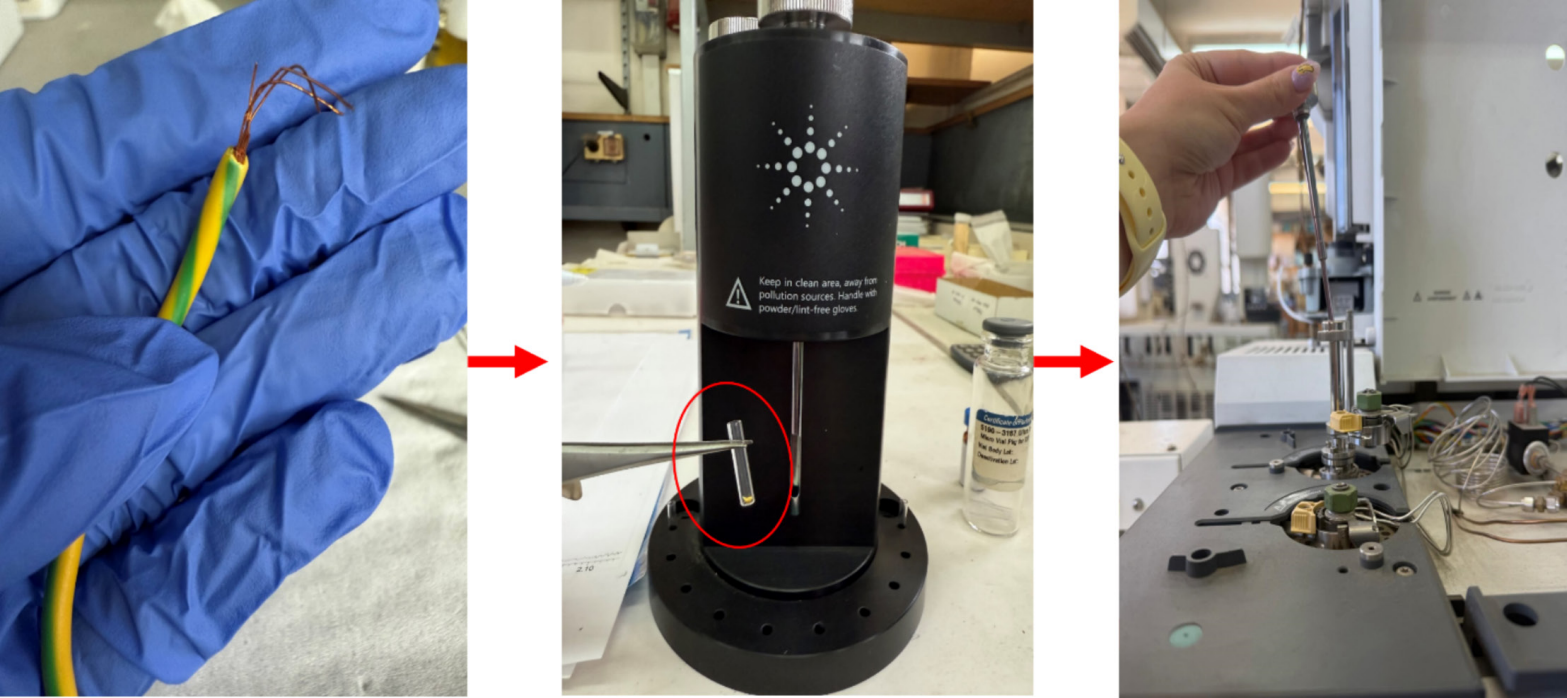
Plastic handling procedure for its phthalate content analysis, as shown in the indicated steps: cutting the desired plastic sample (about or less than 1 mm^3^), placing it inside the ChromatoProbe vial (the photo in the middle), and placing the ChromatoProbe inside the GC inlet for thermal desorption (the photo on the right).

In this study, we used a GC‐MS with Cold EI, that is, a combination of an Agilent (Agilent Technologies, Santa Clara, CA, United States) 7890A GC + 5975B MSD and an Aviv Analytical (Aviv Analytical LTD, Hod Hasharon, Israel) Cold EI, which is based on an SMB interface combined with a dual‐cage fly‐through EI ion source. GC‐MS with Cold EI is discussed in [17, 18, 19, 22, 23], a book about it was published [24], and it was recently reviewed [25]. The output of the GC column is mixed with helium flow rate of 60 mL/min (total for both make up gas and column flow), typically at 830‐mBar pressure and in front of a supersonic nozzle at the end of a temperature‐controlled transfer line. The sample compounds, which are seeded in the helium carrier gas, expand through a 100‐μm diameter supersonic nozzle into a vacuum chamber that is differentially pumped by a Varian Navigator 301 turbo molecular pump (Varian Inc., Torino, Italy) with 250 L/s pumping speed (recently with an Agilent TwisTorr 304 turbo molecular pump). This supersonic expansion results in vibrational cooling of the sample compounds. Moreover, the free jet is collimated and skimmed by a 0.8‐mm skimmer into a second vacuum chamber where an SMB is formed. These vibrationally cold molecules fly‐through a dual‐cage EI ion source and are ionized by 70 eV electrons at an emission current of 6 mA. The generated ions are then focused by an ion lens system, deflected 90° by an ion mirror, and guided into the Agilent 5975B MS for mass analysis and detection with the Agilent triple‐axis ion detector. Data acquisition and processing are performed using Agilent ChemStation software. For tuning of the system and mass calibration, PerFluoroTributylAmine (PFTBA) can be mixed with the helium make‐up gas. The GC was configured with a 15‐m‐length column with 0.32 mm ID, 0.1‐μ DB1HT films, and 4 mL/min column flow rate for 12 min, followed by a flow program of 10 mL/min^2^ up to 24 mL/min with a hold time for 3 min until the end of the analysis. The GC oven was set to 50°C for 0.5 min, then a temperature program of 40°C/min to 350°C with a hold time for 3 min and the transfer line was set to 250°C for 8 min, and then a temperature program of 10°C/min to 280°C with a hold time for 3 min. A standard Agilent Split/Splitless injector was used at 270°C injector temperature in pulsed split mode (25 PSI for 0.7 min), where the split ratio was varied between 3:1 to 9:1 according to the plastic, which was investigated. For sample introduction, the ChromatoProbe was used. Plastic samples were cut into ~1‐mm^3^ fragments (about 0.2–0.3 mg) and placed inside a ChromatoProbe microvial with 2.4‐mm I.D. Then, the ChromatoProbe was placed into the GC inlet for thermal desorption for 0.6 min. Phthalates standard were purchased from LGC Standards (Teddington, United Kingdom) (Product Codes D455385, D455395, and D459900) and analyzed by introducing 2 μL of each sample, measured with a micropipette, into a ChromatoProbe vial. Plastic samples were obtained from office supplies found in the office/lab and were purchased in a stationery store. Figure 1 shows the minimal sample preparation process, which includes cutting the plastic into a small piece, placing it inside the vial of the ChromatoProbe and inserting it into the GC inlet.

## 3 Results and Discussion

**FIGURE 2:**
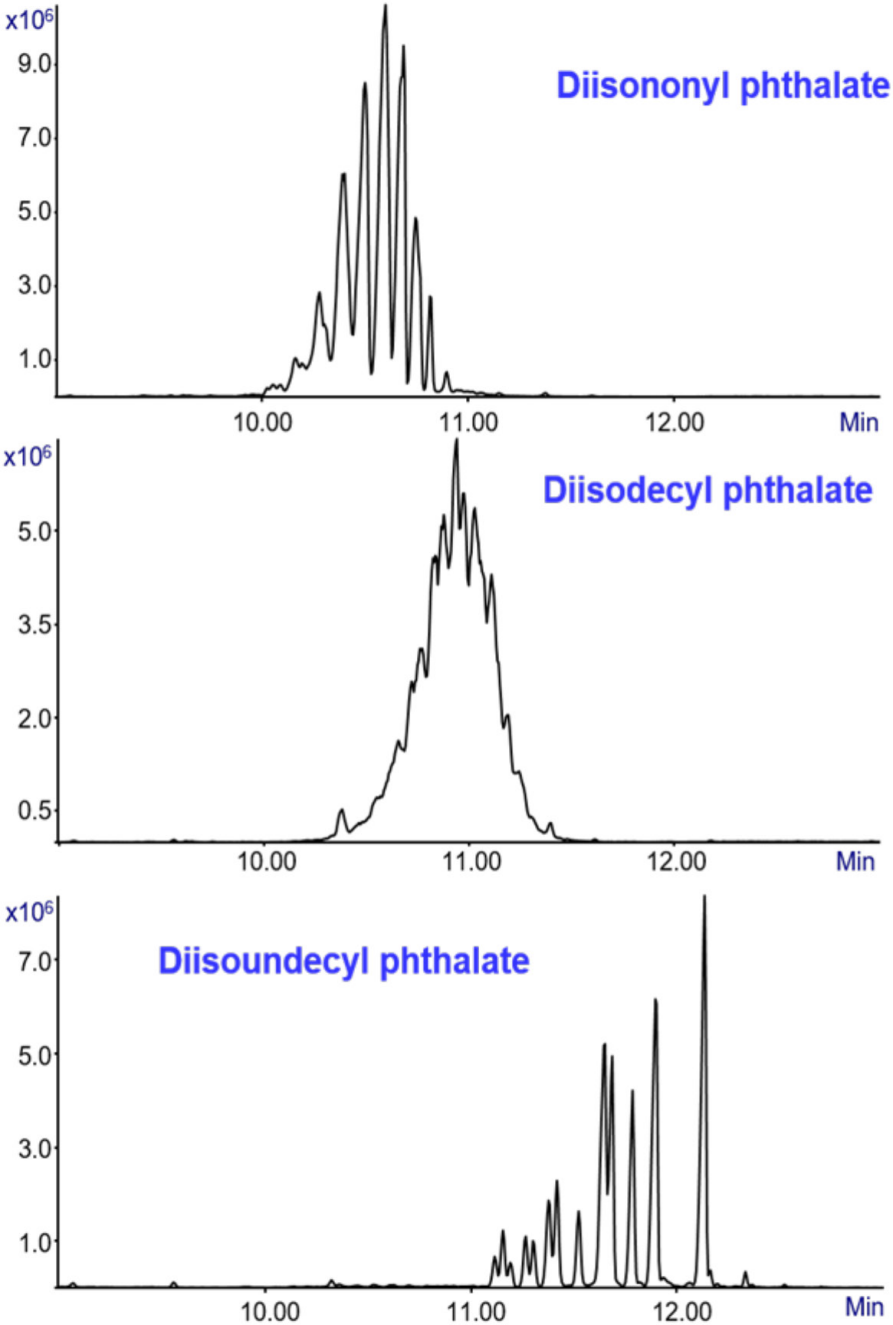
Total ion mass chromatograms (TIC) from GC‐MS with Cold EI analysis of 2 μL injections of 1000 ppm standards in methanol of DINP (top), DIDP (middle), and DIUP (bottom), all dissolved in methanol. Each sample was placed in a ChromatoProbe vial and thermally desorbed into the GC for analysis with a split ratio of 4.

Figure 2 presents three separate analyses, each corresponding to one phthalate standard — DINP, DIDP, and DIUP. All were prepared in methanol at 1000 ppm and analyzed under the same conditions with a 2 μL injection volume and split ratio of 3:1. The figure shows the three ion mass chromatograms of DINP, DIDP, and DIUP with a zoom on their elution times. The chromatographic profiles indicate that these phthalate isomers elute within a similar retention time window, consistent with their structural similarities. A clear correlation between molecular mass and elution behavior is observed. Furthermore, each standard produces a complex chromatographic pattern characterized by multiple peaks, reflecting the presence of numerous structural isomers arising from the mixtures of branched alkyl side chains.

**FIGURE 3:**
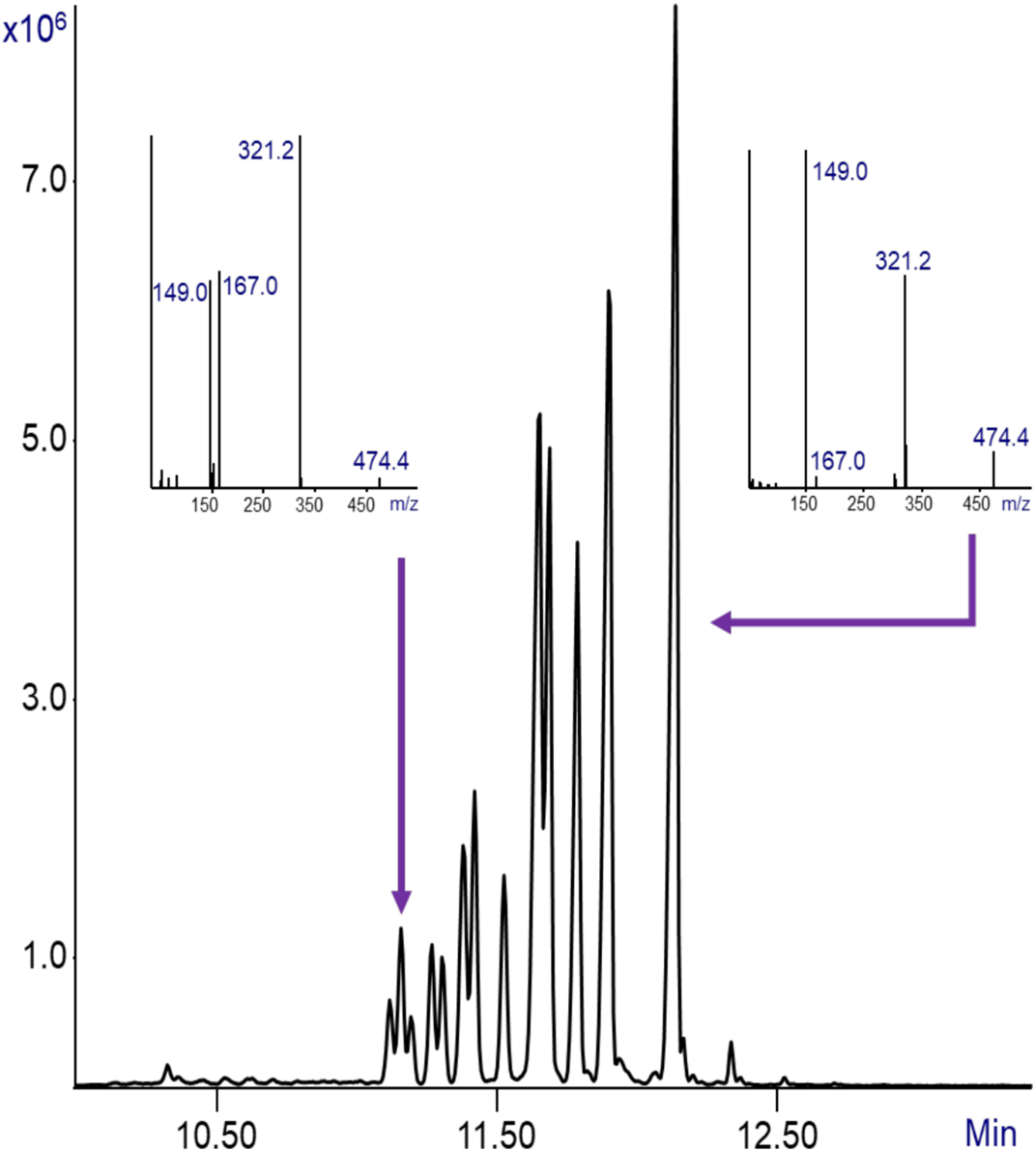
Total Ion mass Chromatogram of 2 μL of a 1000 ppm diundecyl phthalate (DIUP) standard in methanol, as shown previously in Figure 2. Two peaks corresponding to isomers with the molecular ion of m/z = 474.4 are indicated by arrows. Their respective mass spectra are displayed adjacent to the peaks.

Figure 3 shows two representative mass spectra obtained from different isomeric components of the DIUP standard. Although both spectra correspond to the same nominal compound, distinct variations in the fragmentation patterns are evident, reflecting the structural diversity of the branched alkyl side chains within DIUP. Importantly, both spectra exhibit a well‐defined molecular ion at *m*/*z* = 474.4. The presence of this molecular ion is critical for the unambiguous identification of DIUP, because in its absence, the compound cannot be confirmed with certainty based solely on less selective fragment ions. Notably, of the two DIUP isomers, only the later‐eluting isomer (on the right) was correctly identified by the NIST EI mass spectral library as DIUP, whereas the earlier isomer was misidentified as other phthalate (*m*/*z* = 432). This misidentification reflects the high spectral similarity among branched phthalates and the limitations of conventional EI mass spectra in the library, where the molecular ion is often absent. By contrast, Cold EI mass spectra provide clear and stable molecular ions, which resolve such misidentifications and thereby ensure accurate molecular weight determination and support confident identification of the analyte despite the isomeric complexity of the mixture. In addition, note the good chromatographic peak shapes due to the elimination of ion source‐related peak tailing with Cold EI.

**FIGURE 4:**
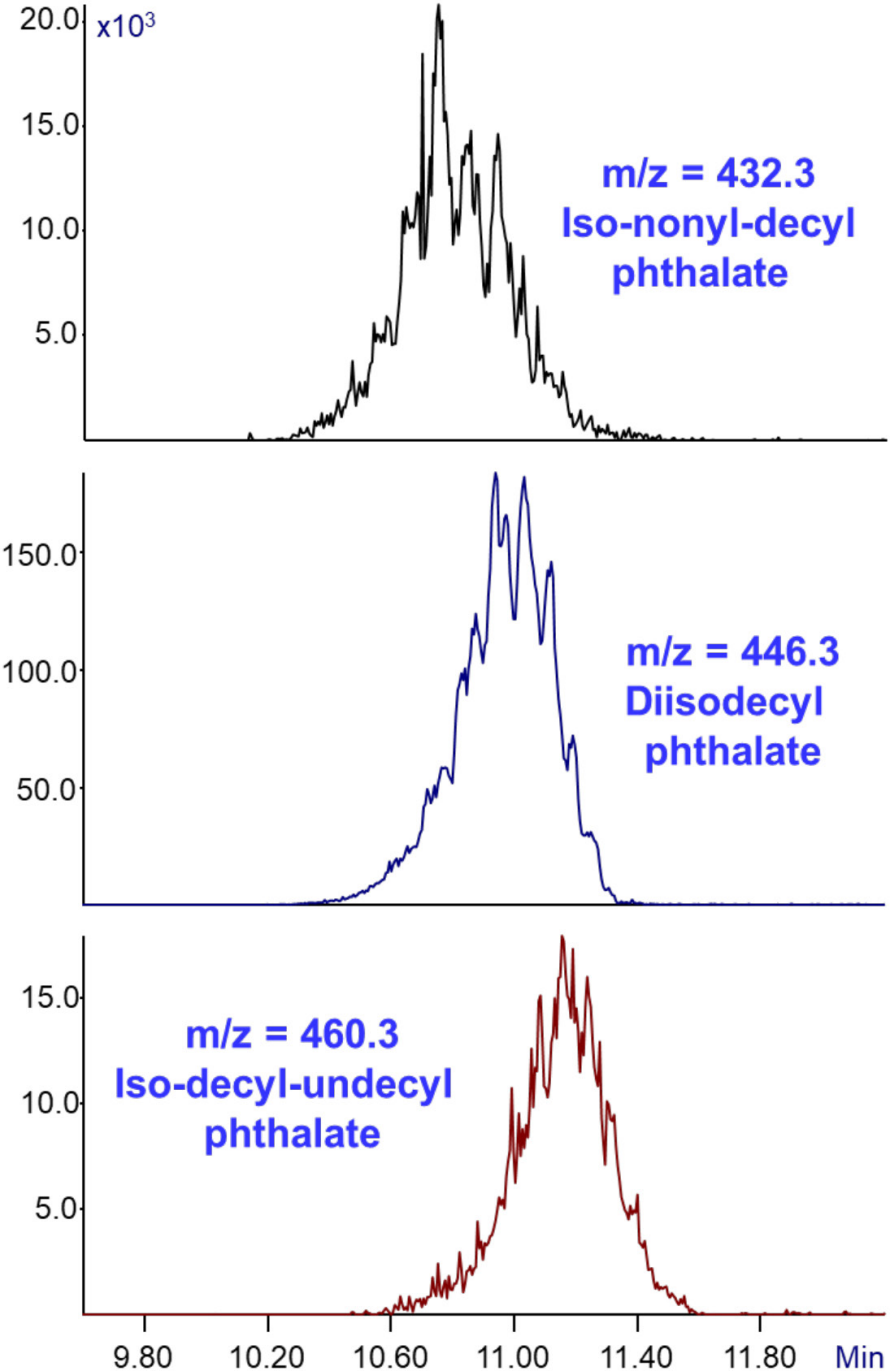
Extracted ion mass chromatograms of a diisodecyl phthalate (DIDP) standard acquired at m/z = 432.3, 446.3, and 460.3 (top to bottom) using GC‐MS with Cold EI. The data demonstrates how Cold EI can uniquely detect the presence of mixed alkyl phthalates (iso‐nonyl‐decyl phthalate and Iso‐decyl‐undecyl phthalate) within the DIDP standard and provide their relative concentration as shown on the *Y* axis.

Figure 4 presents the extracted ion mass chromatogram of the analysis of DIDP (for *m*/*z* = 432.3, 446.3, and 460.3) as shown previously in Figure 2. As can already be observed in the mass chromatogram of DIDP in Figure 2, the standard does not consist of a single compound but rather exhibits numerous peaks corresponding to different isomers. This observation led us to assume that the mixture might also contain mixed alkyl phthalates in addition to the isodecyl isomers. To investigate this, masses differing by ± CH_2_ units relative to the molecular ion were monitored in order to detect potential mixed alkyl species. The extracted ion mass chromatogram traces highlight *m/z* values of 432.3, 446.3, and 460.3, corresponding to iso‐nonyl‐decyl phthalate, diisodecyl phthalate, and iso‐decyl‐undecyl phthalate, respectively. These compounds were identified as the most abundant phthalates present in the DIDP standard.

Importantly, the mixed alkyl phthalates detected exhibited an abundance of approximately 10% or a little more relative to DIDP, a reliable estimate made possible because GC‐MS with Cold EI is characterized by a uniform response across different analytes. The presence of mixed alkyl phthalates in a commercial DIDP standard confirms that these compounds are integral to DIDP mixtures and consequently should be anticipated in consumer products manufactured with DIDP, which has direct implications for regulatory monitoring. Moreover, the presence of these additional phthalates raises concerns about human exposure, as mixtures rather than pure compounds are typically encountered in real‐world settings.

**FIGURE 5:**
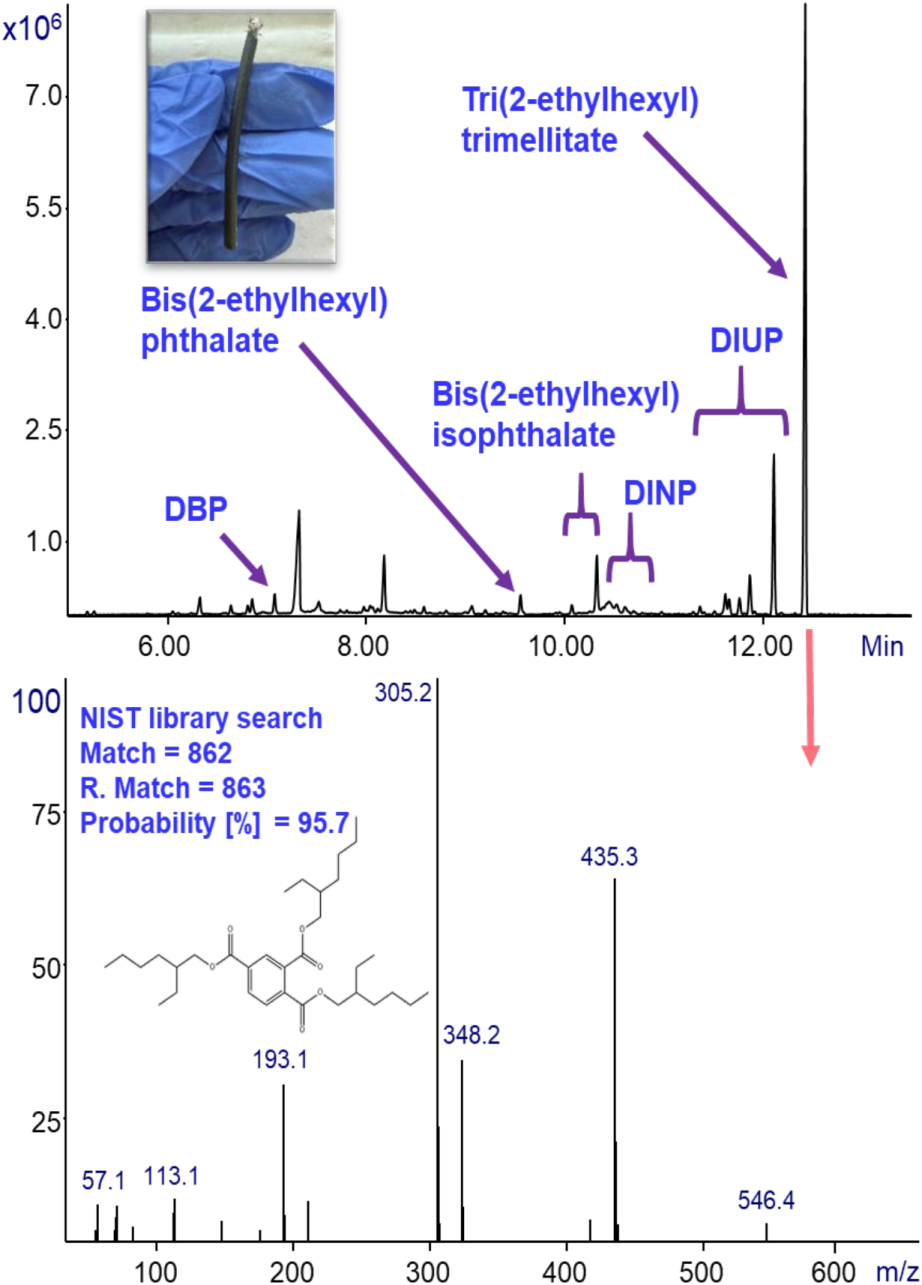
GC‐MS with Cold EI analysis of a small piece (below 1 mm^3^) of a black electricity cable using ChromatoProbe for sample introduction and thermal desorption. The total ion mass chromatogram is shown on top with the names of the phthalates identified marked with an arrow. At the bottom, the mass spectrum of the last eluting peak, which is a heavy phthalate (*m*/*z* = 546.4), is presented, along with its identification parameters from the NIST EI mass spectral library and corresponding structural formula.

**FIGURE 6:**
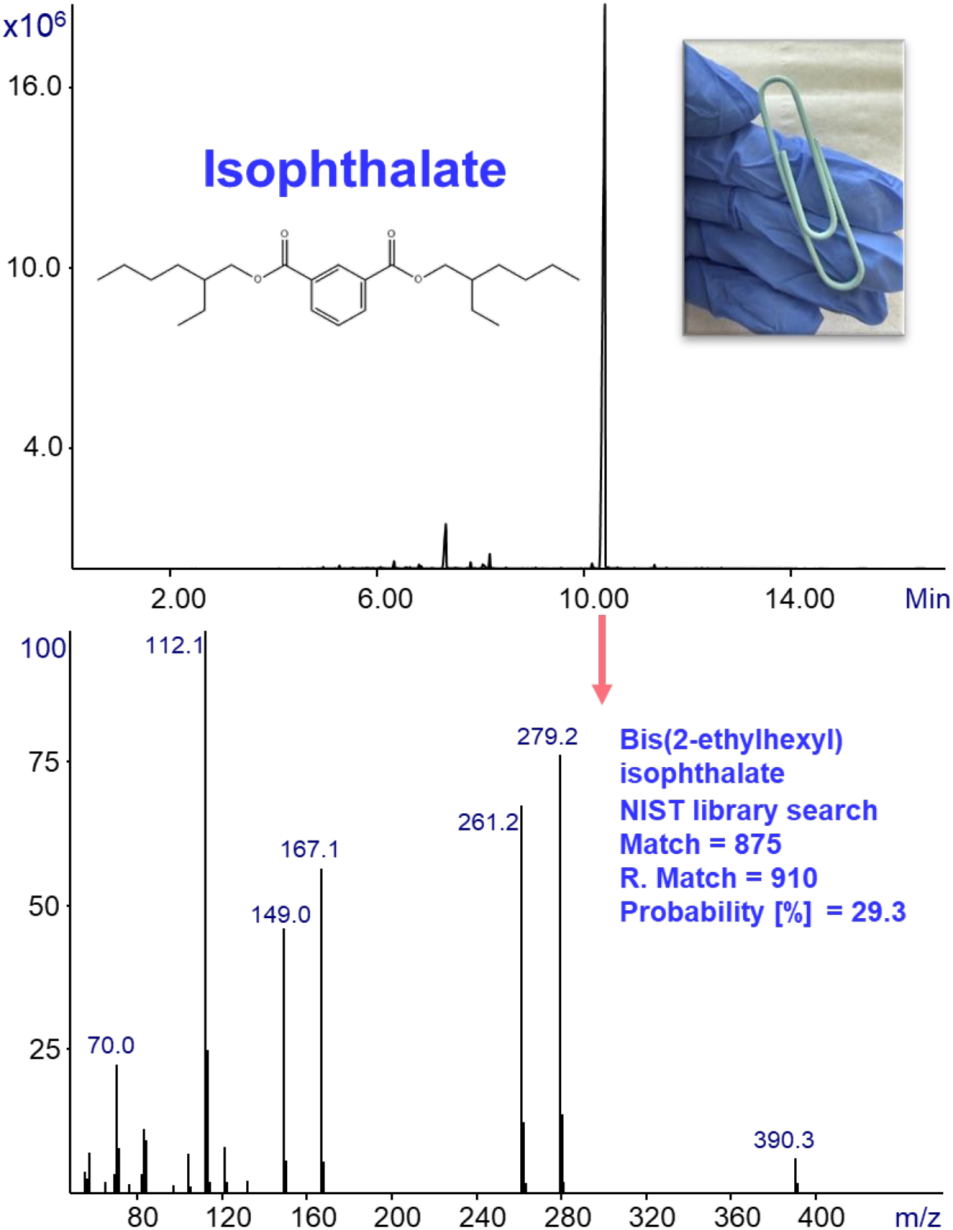
GC‐MS with Cold EI analysis of a small piece (under 1 mm^3^) of a paper clip using ChromatoProbe for sample introduction and thermal desorption. The total ion mass chromatogram is shown on the top, with the most abundant peak annotated with its group name (isophthalate) and structural formula. The corresponding mass spectrum is shown at the bottom, including the compound's identification parameters from the NIST EI mass spectral library.

**FIGURE 7:**
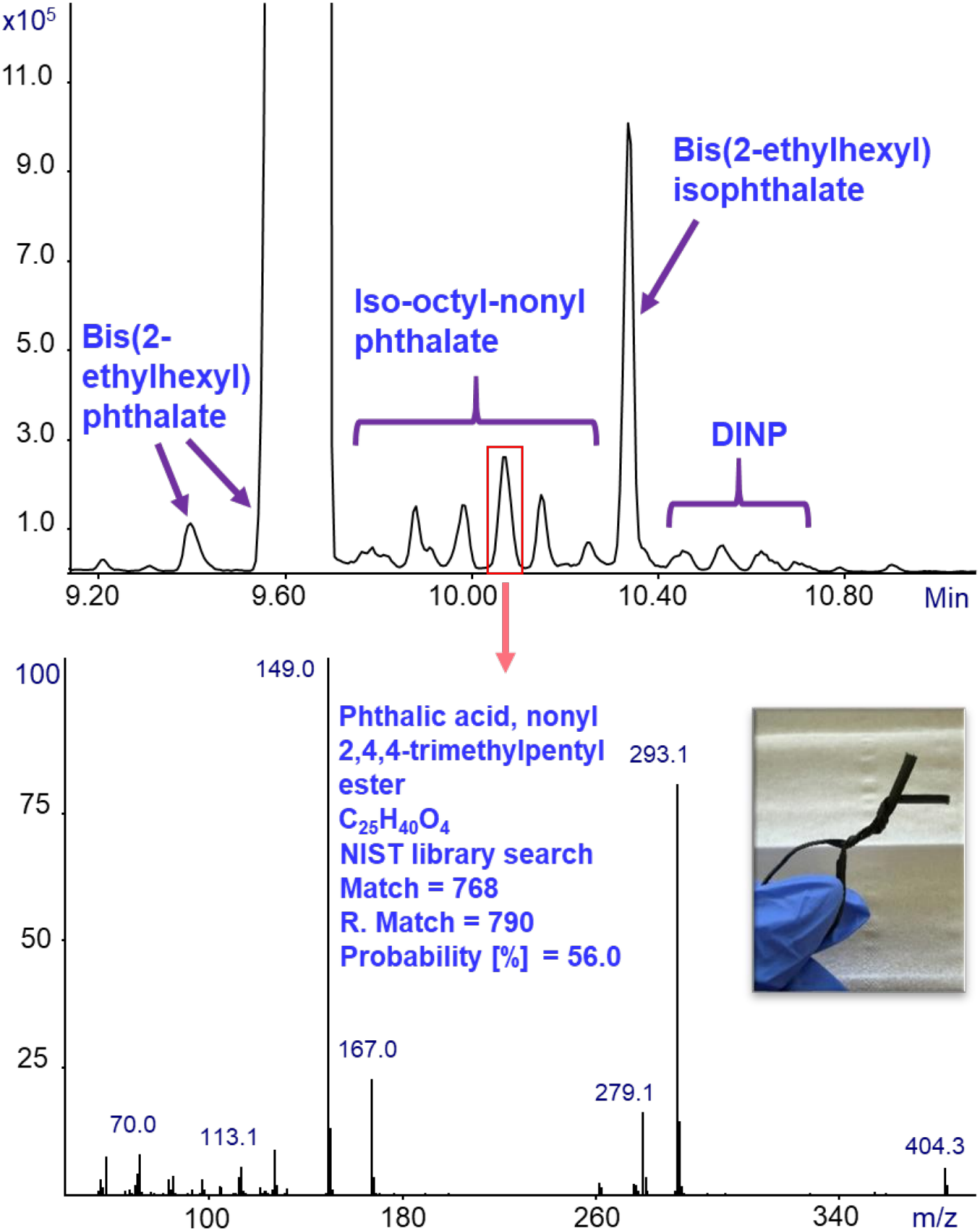
GC‐MS with Cold EI analysis of a small piece (below 1 mm^3^) of a twist tie using ChromatoProbe for sample introduction and thermal desorption. The total ion mass chromatogram is shown on the top with the names of several phthalates identified marked with an arrow. The mass spectrum of the marked peak, which is a mixed alkyl phthalate, is shown at the bottom, including the compound's identification parameters from the NIST EI mass spectral library.

To evaluate the phthalates composition in plastics, we investigated a few products found in our lab, and Figures 5, 6, 7 present the results of their analysis. Figure 5 shows the analysis of a black electricity cable commonly used in our laboratory (for 220‐V AC voltage input).

The mass chromatogram reveals a complex profile with numerous peaks corresponding to a wide range of phthalates, spanning from lower‐mass species such as dibutyl phthalate (*m*/*z* = 278) to higher mass plasticizers including DINP and DIUP (*m*/*z* = 418 and 474). Phthalates that were detected and identified through NIST EI mass spectral library are marked in the mass chromatogram with arrows and their corresponding names. Most notably, the last‐eluting peak was identified as tri‐2‐ethylhexyl trimellitate, an unusually heavy phthalate (*m*/*z* = 546). This represents a new and unexpected finding, as this heavy phthalate is not commonly reported in such materials. Its mass spectrum, shown at the bottom of the figure together with the structural formula and the NIST identification parameters, exhibits a high library identification probability of 95.7%. Importantly, the mass spectrum displays a clear molecular ion, which enabled confident identification using GC‐MS with Cold EI mass spectra.

Another representative analysis is shown in Figure 6 of a paper clip (plastic‐coated metal fastener for holding paper sheets) found in our office. The ion mass chromatogram of the paper clip is shown at the top of the figure, and the Cold EI mass spectrum of the arrowed peak with its NIST identification parameters is presented at the bottom. Unlike the electricity cable analysis, the paper clip displays a much simpler mass chromatogram, characterized by far fewer peaks and essentially only a single dominant peak at ~10 min. This indicates that, unlike the previous sample that contained multiple phthalates, the paper clip contains only one major plasticizer. NIST library identification confirmed this peak as dioctyl isophthalate (DIOP), an isomer of the widely used plasticizer and most commonly used commercial compound — dioctyl phthalate (DOP or DEHP) [11]. The distinction between the two lies in the position of the ester substituents on the aromatic ring (ortho in DOP vs. meta in DIOP).

Generally, ortho‐phthalates (such as DBP, DOP, and DINP) are more commonly used than isophthalate species (such as DIOP) mainly because of their low cost, availability, and high plasticization efficiency [26]. On the other hand, the isophthalates can be used to bypass the restrictions, as they are considered to be less toxic. Their different geometry also produces a distinct EI mass spectrum, enabling clear differentiation between the isomers. Importantly, the ability to clearly observe the molecular ion and distinguish between such isomers demonstrates the strength of GC‐MS with Cold EI in providing unambiguous identification of plasticizers in complex polymeric samples. Figure 7 presents the analysis of a small plastic piece used as a twist tie (a flexible fastening wire). The mass chromatogram, shown at the top, contains multiple phthalates detected and identified using the NIST EI mass spectral library, which are marked with arrows and their corresponding names.

**FIGURE 8:**
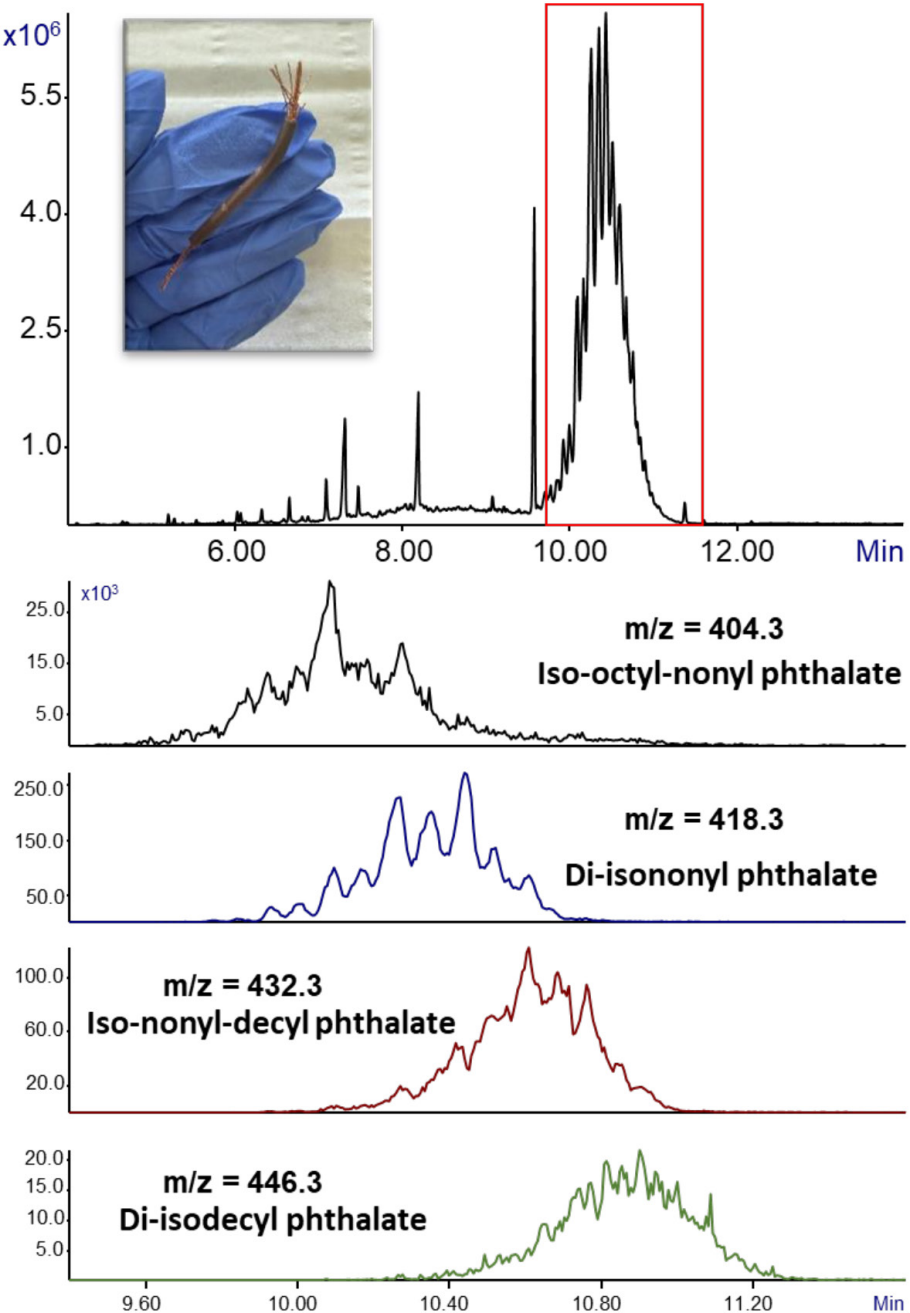
GC‐MS with Cold EI analysis of a small piece (< 1 mm^3^) of a brown electricity cable using ChromatoProbe for sample introduction and thermal desorption. The total ion mass chromatogram is shown on the top. The extracted ion mass chromatograms for *m*/*z* = 404.3, 418.3432.3, 446.3, and 446.3 — corresponding to various phthalates and mixed alkyls — are shown at the bottom, highlighting the peak within the red box. As shown in the figure, the extended range of GC‐MS with Cold EI and its enhanced molecular ions enables the detection and identification of the different phthalates inside a mixture.

The EI mass spectrum of the arrowed peak, together with its NIST identification parameters, is presented at the bottom. This analysis reveals that the twist tie contains several phthalates, including the widely used DOP (major peak), its isomer DIOP, the high‐volume plasticizer DINP, and a mixed alkyl phthalate — iso‐octyl‐nonyl phthalate. The presence of both common phthalates and mixed alkyl phthalates demonstrates the tendency of manufacturers to employ blends of plasticizers, likely to balance cost and performance. Importantly, without the clear molecular ion at *m*/*z* = 404.3 provided by GC‐MS with Cold EI, the mixed alkyl phthalate could have been misidentified (or not been identified at all) if based solely on its fragment ions at *m*/*z* = 149 and 293, which are nonselective and shared by multiple phthalates. This figure therefore illustrates not only the chemical complexity of plastic consumer products but also emphasizes the critical advantage of GC‐MS with Cold EI in generating mass spectra with strong molecular ions that enable the confident and unambiguous identification of individual phthalates within complex mixtures. Figure 8 presents the analysis of another electricity cable (brown). The total ion mass chromatogram, shown at the top of the figure, exhibits numerous peaks, including a major broad unresolved cluster of peaks around 10–12 min, similar to the behavior observed previously for DIDP (Figure 2). Such chromatographic behavior is characteristic of mixed alkyl phthalates. To further investigate, extracted ion mass chromatograms were applied to selectively monitor the expected mixed alkyl molecular ions. As shown, it reveals that this wire contains predominantly DINP, along with additional mixed alkyl species including iso‐nonyl‐decyl phthalate, iso‐octyl‐nonyl phthalate, and a smaller contribution of DIDP. Importantly, the clear molecular ion signals provided by Cold EI enabled the confident detection and differentiation of these overlapping mixed alkyl phthalates, even within a highly complex chromatographic region. Notably, comparison of this brown wire with the previously analyzed electricity cable demonstrates that two wires with similar appearance and common use in our laboratory can exhibit completely different plasticizer compositions.

**FIGURE 9:**
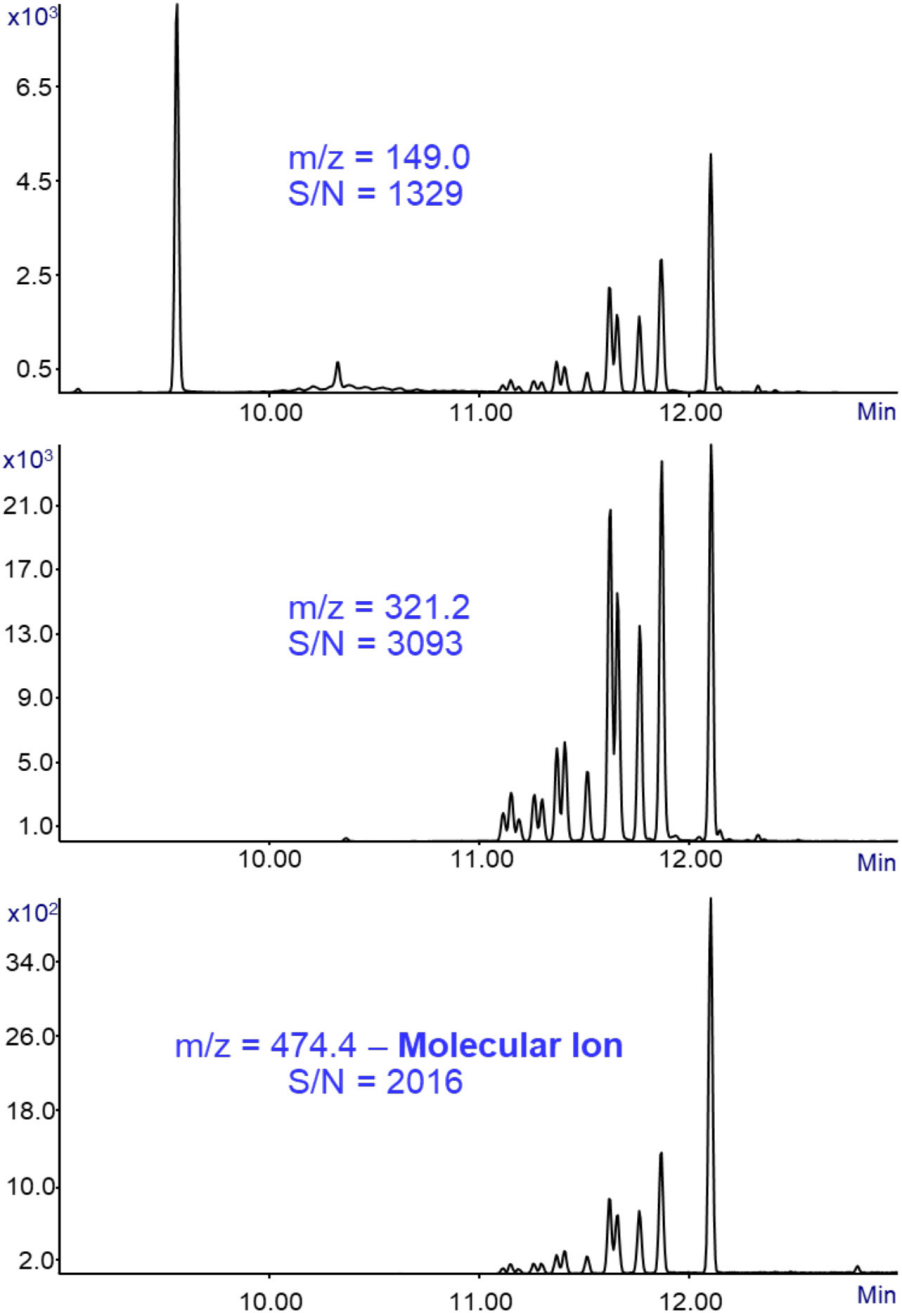
Sensitivity evaluation. A mass chromatogram of 10 pg (on column) diundecyl phthalate standard in methanol analyzed in three analyses in SIM (selective ion monitoring) mode for *m*/*z* = 149.0, 321.2, and 474.4 (respectively top to bottom) with a dwell time of 300 ms each. Each chromatogram shows the signal‐to‐noise ratio (as peak‐to‐peak) for the corresponding *m*/*z* value (note that the on‐column amount of each isomer is about 2 pg; thus, the extrapolated LOD is approaching 1 fg).

Sensitivity is a critical parameter in GC‐MS analysis, as it determines the ability to detect low concentrations of analytes and directly impacts the reliability of identification in complex samples. To evaluate this aspect, Figure 9 presents three analyses of 10 pg (on column) of DIUP in SIM (selective ion monitoring) mode, monitoring the fragment ions at *m*/*z* = 149.0 and 321.2 along with the molecular ion at *m*/*z* = 474.4 with a dwell time of 300 ms, and each chromatogram shows the signal‐to‐noise ratio of its *m*/*z*. Remarkably high signal‐to‐noise ratios were obtained for all three monitored ions, demonstrating the outstanding sensitivity of the GC‐MS with Cold EI system and its capability to detect DIUP even at trace‐level concentrations with minimal background interference.

Notably, DIUP exhibits a molecular ion of zero or only ~0.2% relative abundance in the NIST EI mass spectral library, meaning that under standard EI conditions the molecular ion is practically absent and sensitivity evaluation based on it would not be feasible. In contrast, GC‐MS with Cold EI mass spectrum of DIUP provides a strong and distinct molecular ion at *m*/*z* = 474.4, enabling accurate sensitivity assessment and S/*N* = 2016 (peak‐to‐peak noise), confirming its superior performance for trace‐level analysis because the molecular ion is far more selective than fragment ions.

Another major aspect of phthalate analysis is quantitative determination. As some phthalates are restricted to 0.1% (w/w) in plastics, accurate quantification is sometimes of critical importance. To evaluate the quantitative performance of Cold EI, two analyses were performed: a paper clip sample and a DINP standard in methanol (1000 ppm), used as a reference. Initial analysis of the paper clip showed that its phthalate content (predominantly DINP) was not fully thermally desorbed, as the same piece could be reanalyzed with high residual phthalate content. This observation indicates that phthalates diffuse out of the inside of the plastic matrix rather than being instantaneously thermally desorbed. Consequently, the paper clip was cut into a smaller piece weighing only 80 μg. In addition, the thermal desorption time in the inlet was extended to 6 min, and the GC oven temperature program was modified by holding the oven at 50°C for the first 6 min. The same plastic sample was then analyzed twice in consecutive runs. As Cold EI provides a uniform response, DINP signals from the first and second analyses were compared. Only ~0.4% of DINP was detected in the second analysis, indicating that nearly all DINP was thermally desorbed during the first run. For DINP quantification, the standard reference was analyzed under the same conditions using a 2 μL injection. Based on the quantitative analysis performed, the measured DINP content was 10.0% (8.0 μg) of the paper clip mass, consistent with literature reports indicating that phthalates may constitute up to 40% (w/w) of plastic materials [27]. These results demonstrate that the uniform response of Cold EI enables accurate quantification of heavy phthalates in complex plastic matrices.

## 4 Conclusions

This paper describes an analytical method and instrumentation for the analysis of plastics for their phthalate content, using ChromatoProbe sample introduction to eliminate any sample preparation and GC‐MS with Cold EI for improved performance. The developed approach demonstrates several key benefits and achievements:

1. GC‐MS with Cold EI enables the detection and improved identification of a wide range of phthalates — from low‐molecular‐weight (*m*/*z* = 194) to heavy‐molecular‐weight (*m*/*z* = 546) and mixed alkyl species, through the consistent observation of their molecular ions. This capability allows reliable identification of compounds that are often misidentified or remain undetected using GC‐MS with standard EI for analysis.
2. GC‐MS with Cold EI provides clear molecular‐ion information that enables the differentiation between structural isomers such as DOP and DIOP. The ability to distinguish these isomers based on their distinct mass spectra highlights the selectivity and the reliable obtaining of molecular ions achieved with GC‐MS with Cold EI.
3. GC‐MS with Cold EI allows the characterization and relative quantification of mixed alkyl phthalates (such as iso‐octyl‐nonyl phthalate) by providing a distinct molecular ion that prevents misidentification based on nonselective fragment ions that indicate an incorrect single‐alkyl phthalate rather than the mixed alkyl phthalate. This capability is crucial for the correct interpretation of complex plastics containing multiple overlapping phthalates, which generate similar fragment ions when analyzed with GC‐MS with standard EI. The use of GC‐MS with Cold EI further facilitated the reliable detection of these mixed alkyl phthalates by providing enhanced molecular ions that are absent in standard EI spectra, thereby enabling accurate recognition of species that might otherwise be overlooked, even within commercial standards.
4. GC‐MS with Cold EI exhibits outstanding sensitivity and stability, as demonstrated by the high signal‐to‐noise ratios obtained for DIUP (at a 10 pg on‐column amount). This observation demonstrates its superiority for trace‐level analysis and quantitative evaluation of plasticizers, even in challenging analytical conditions.

Beyond the analytical achievements demonstrated, the results reveal several broader implications related to phthalate composition, manufacturing trends, and potential applications of this analytical approach:

- We detected phthalates in all the plastic samples that we analyzed, mostly dioctyl phthalate and its isomers.
- Several plastic samples were found to contain multiple phthalates within a single material, revealing the compositional complexity of common plastic products. For instance, the analysis of a black electricity cable demonstrated the presence of up to six different phthalates, including mixed alkyl species. Such diversity within one sample may result from the use of recycled materials, cross‐contamination during manufacturing, or intentional blending of plasticizers to achieve specific mechanical or thermal properties. This finding underscores the importance of comprehensive analytical approaches, such as Cold EI, which can reliably detect and differentiate multiple coexisting phthalates and mixed alkyl plasticizers within complex plastic matrices.
- The detection of tri‐2‐ethylhexyl trimellitate in plastic samples suggests that manufacturers may be shifting toward heavier, currently unregulated phthalates as substitutes for restricted compounds, as their impact on human health remains uncertain [28].
- The detection of DIOP in a plastic sample, rather than the more common DOP, likely reflects a deliberate substitution by manufacturers. Although DOP has historically been the most efficient and widely used PVC plasticizer, its use is now heavily regulated and restricted due to health concerns, particularly in children's products, medical devices, and food‐contact materials. In contrast, DIOP, though less efficient, is not subject to the same level of restriction, making it an appealing alternative in certain consumer products.
- In this study, the results demonstrate that GC‐MS with Cold EI is also well suited for phthalate quantification, even in complex matrices, as supported by the quantitative analysis of DINP in a plastic sample. The generation of strong and stable molecular ions enables correct compound identification and provides a uniform response across different analytes. In contrast, GC‐MS with standard EI often results in misidentification and quantification errors, particularly in the analysis of heavy phthalates, because the absence or weakness of their molecular ions forces identification to rely on common fragment ions shared by multiple phthalates and mixed alkyl phthalates. Consequently, mixed alkyl phthalates may be misidentified as two separate single‐alkyl phthalates (e.g., DINP and DOP), because the lack of a molecular ion forces identification to depend on nonspecific fragment ions shared among structurally related compounds. Moreover, under standard EI conditions, heavy phthalates frequently exhibit peak tailing, leading to nonlinear detection responses that further compromise quantification accuracy. Such limitations highlight the importance of reliable molecular‐ion generation for accurate quantitative analysis, especially for regulated compounds such as DINP, which is restricted to concentrations below 0.1% in certain consumer products.
- The results also suggest the potential of phthalate profiling for forensic and comparative applications. Because each plastic product has a characteristic phthalate composition that reflects its formulation and manufacturing process, this information can serve as a chemical fingerprint for material identification. The ability of GC‐MS with Cold EI to detect a broad range of phthalates — including heavy and mixed alkyl species — enhances the discriminatory power of such analyses, allowing the differentiation of plastics with similar appearance or function. Consequently, phthalate composition could be used to trace the origin, production source, or intended use of a plastic item in forensic investigations, quality control, and counterfeit detection.

GC‐MS with Cold EI is a highly superior technology for phthalates analysis; however, it is not yet commercially available as a routine analytical approach. Moreover, the addition of the SMB interface and the dual‐cage fly‐through ion source can introduce a modest increase in system cost compared to GC‐MS with standard EI. In addition, GC‐MS with Cold EI consumes more helium per minute compared to GC‐MS with standard EI as it is being used for both carrier and make‐up gas as described in our study. The use of a high column flow rate of helium results in a much faster analysis compared to a standard GC‐MS analysis; thus, the total helium usage remains comparable. Furthermore, a key advantage of GC‐MS with Cold EI is that it can be operated without helium, using hydrogen or nitrogen as carrier and make‐up gasses, as described in [29].

Overall, this study demonstrates the analytical power and reliability of GC‐MS with Cold EI and ChromatoProbe for comprehensive, sample preparation‐free characterization of phthalate plasticizers in plastics, establishing it as a robust tool for both research and regulatory applications.

## Funding

This work was supported by the Israel Science Foundation (312/22).

## Data Availability

The data that support the findings of this study are available from the corresponding author upon reasonable request.
